# Sorption of Hg(II) and Pb(II) Ions on Chitosan-Iron(III) from Aqueous Solutions: Single and Binary Systems

**DOI:** 10.3390/polym10040367

**Published:** 2018-03-24

**Authors:** Byron Lapo, Hary Demey, Jessenia Zapata, Cristhian Romero, Ana María Sastre

**Affiliations:** 1 School of Chemical Engineering, Universidad Técnica de Machala, UACQS, BIOeng, 070151 Machala, Ecuador; jmzapata_est@utmachala.edu.ec (J.Z.); caromeros_est@utmachala.edu.ec (C.R.); 2 Department of Chemical Engineering, Universitat Politècnica de Catalunya, EPSEVG, Av. Víctor Balaguer, s/n, 08800 Vilanova i la Geltrú, Spain; 3 Commissariat à l’Energie Atomique et aux Energies Alternatives, CEA/DRT/LITEN/DTBH/LTB, 17 rue des Martrys, 38054 Grenoble, France; 4 Department of Chemical Engineering, Universitat Politècnica de Catalunya, ETSEIB, Diagonal 647, 08028 Barcelona, Spain; ana.maria.sastre@upc.edu

**Keywords:** binary, chitosan, desorption, iron, lead, mercury, salt effects, single, sorption competition

## Abstract

The present work describes the study of mercury Hg(II) and lead Pb(II) removal in single and binary component systems into easily prepared chitosan-iron(III) bio-composite beads. Scanning electron microscopy and energy-dispersive X-ray (SEM-EDX) analysis, Fourier transform infrared spectroscopy (FTIR), thermogravimetric analysis (TGA) and point of zero charge (pH_pzc_) analysis were carried out. The experimental set covered pH study, single and competitive equilibrium, kinetics, chloride and sulfate effects as well as sorption–desorption cycles. In single systems, the Langmuir nonlinear model fitted the experimental data better than the Freundlich and Sips equations. The sorbent material has more affinity to Hg(II) rather than Pb(II) ions, the maximum sorption capacities were 1.8 mmol·g^−1^ and 0.56 mmol·g^−1^ for Hg(II) and Pb(II), respectively. The binary systems data were adjusted with competitive Langmuir isotherm model. The presence of sulfate ions in the multicomponent system [Hg(II)-Pb(II)] had a lesser impact on the sorption efficiency than did chloride ions, however, the presence of chloride ions improves the selectivity towards Hg(II) ions. The bio-based material showed good recovery performance of metal ions along three sorption–desorption cycles.

## 1. Introduction

Heavy metals are potentially toxic to humans; mercury and lead are two of the most harmful metals present in wastewater [1], some studies label these metals as relevant and very toxic elements [2,3]. Even nowadays, the presence of these metals, particularly in water resources is reported [4,5], hence it represents a potential risk to our ecosystems. Typical ways of being released into the environment are mining, smelters, coal burning, hydropower plants, agriculture system, etc. [6], particularly, these contaminants can reach the natural waters degrading its quality. Nevertheless, the scientific community is constantly developing removal methods, such as adsorption, membrane filtration, electrodialysis, ionic liquids [7,8], towards a suitable solution to this problem.

Sorption and particularly biosorption are some of the most promising methods to remove toxic metals. Particular focus is given to innovative adsorbents based on biomaterials. One of the major potential substances is the chitosan, a well-known biopolymer derived from alkaline *N*-deacetylation of chitin [9], which can surpass its natural limitations in aqueous media throughout targeted modifications, driving to enhance its adsorption properties as well as its selectivity for targeted ions and mechanical properties. Several chitosan-based materials have been reported to provide excellent sorption of Hg(II) and Pb(II) [10,11,12,13]. Although, many reported chitosan-composite materials have shown excellent performance in terms of sorption capacity, several limitations make the industrial scaling-up difficult. Some restraints are: Very low particle size, which can produce blockage in hydraulic systems, low mechanical stability that does not enable the use in columns, extended use of chemicals for chitosan matrix modification, not to mention in many cases the related toxicity of the chemical compounds used is overlooked.

Iron(III) represents an alternative to making this possible. It presents many advantages such as being abundant, cheap and easy to manage. It could also provide better mechanical properties to chitosan. However, the major studies are based on magnetic iron, which involves complex procedures and high energy consumption to achieve the desired magnetic properties. Nevertheless, few researchers have proposed chitosan-iron composite without further process in adsorbing metals and metalloids from water. Example of these are: As(III) and As(V) with Fe(III) immobilized on chitosan beads [14], and boron from seawater with chitosan iron(III) hydroxide beads [15]. However, two of the most toxic and highly relevant heavy metals, Hg(II) and Pb(II) have not been studied with this promising bio-based material.

On the other hand, the major of studies are targeted toward single element studies, neglecting the synergic effect of other ions in real water, which is definitively important for assessing industrial wastewater treatment. Focusing on developing a potential material with industrial insight, which means the convergence between cost, good sorption–desorption performance and being environmentally friendly, the present study targets a single, binary component, desorption, salt interference on the sorption of Hg(II) and Pb(II) using beads based on chitosan-iron(III) composite.

## 2. Materials and Methods

### 2.1. Chemicals

Chloride(II) nitrate (HgCl_2_, 99%, (Probus, Barcelona, Spain), lead(II) nitrate (Pb(NO_3_)_2_; 98%, Panreac, Barcelona, Spain), chitosan (Aber Technologies, Lannilis, France, M_W_ = 125,000 g·mol^−1^ was determined by gel permeation chromatography technique, and the degree of acetylation DA = 0.13 was obtained by Fourier transform infrared spectroscopy [16,17]), acetic acid (CH_3_COOH, 99.7%, Panreac, Barcelona, Spain), hydrochloric acid (HCl, 37.4%, J.T. Baker, Phillipsburg, NJ, USA), sodium hydroxide (NaOH, 97%, Probus, Barcelona, Spain), Sodium Borohydride (NaBH_4_, ≥96%, Sigma-Aldrich, St. Louis, MO, USA), nitric acid (HNO_3_, 64.9%, Phillipsburg, NJ, USA), sodium sulfate (Na_2_SO_4_, 99.5%, Prolabo, Fontenay-sous-Bois CEDEX, France), sodium chloride (NaCl, 99.5%, Prolabo, Fontenay-sous-Bois CEDEX, France), Iron(III) chloride (FeCl_3_·6H_2_O, 99–102%, Fluka, Buchs, Switzerland, Thiourea (SC(NH_2_)_2_, 99%, Panreac, Barcelona, Spain), Ethylenediaminetetraacetic acid disodium salt dihydrate (EDTA, 99% Panreac, Barcelona, Spain) and deionized water type II laboratory water were used.

### 2.2. Preparation of Composite Beads

The composite beads were prepared with a slight improvement on our previous sorbent material reported by Demey et al. [15]. Neat chitosan was dissolved in acetic acid (1% *w/w*). Parallelly, a previously prepared solution of FeCl_3_·6H_2_O (30% *w/w*) was mixed with chitosan solution. Then, the mixture was homogenized for 2 h at 600 rpm. This solution was added drop-by-drop into a solution of NaOH 1 M through a thin nozzle (Ø 2.0 mm), assisted by a peristaltic pump. For the beads manufacturing, the mixture was first washed with high quantities of type II laboratory water to remove the excess of iron, and dried in a laboratory freeze drier (LyoQuest-55, Telstar equipment, São Paulo, Brazil) at 218 K and 0.05 mbar.

### 2.3. Characterization

Infrared spectrum was performed from 450 to 4000 cm^−1^ in a FTIR Thermo Scientific Nicolet 6700 (Madison, WI, USA); the samples were crushed and blended with potassium bromide (KBr) (2 mg of material in 100 mg of KBr) to make the pellets, prior to FTIR analysis. Thermogravimetric analysis was carried out with a TGA/SDTA 851e/LF/1100 thermobalance (Mettler Toledo, Mississauga, ON, Canada). Samples with mass of 6 mg were degraded between 30 and 800 °C at a heating rate of 10 °C·min^−1^ in N_2_ atmosphere. The pH_pzc_ was evaluated according to the methodology of Yazdani et al. [18] with a Bante 901 Benchtop pH meter instruments (Bante, Shanghai, China). The morphological observations, and energy dispersive X-ray (EDX) probe analysis of composite beads was done before and after metal sorption, using a Phenom XL SEM-EDX (PhenomWorld, Rotterdam, The Netherlands).

### 2.4. pH Study

The optimum pH to perform the sorption experiments was evaluated prior to the equilibrium, kinetics, salt effects and desorption studies. In 25 mL of separated solutions of 0.2 mmol·L^−1^ Hg(II), 0.2 mmol·L^−1^ Pb(II), and 0.15 mmol·L^−1^ of mixed [Hg(II)-Pb(II)] solutions with adjusted pH of 2.0, 3.0, 3.5,4.0, 4.5, 5.0, 5.5 and 6.0 were added to around 20 mg of adsorbent material. After 48 h of agitation (180 rpm), the initial and the final pH values were recorded. Solutions of diluted HNO_3_ and NaOH were used to conveniently adjust the pH. The analysis of Hg(II) was carried out using an atomic absorption spectrophotometer (AAS) Shimazdu AA6300 (Shimadzu Corporation, Kyoto, Japan), equipped with a hydride vapor generator, and the Pb(II) analysis was carried out in an Agilent Technologies 4100 microwave plasma atomic emission spectrometer (MP–AES) (Agilent Technologies, Melbourne, Australia). The sorption capacity (*q*_e_) versus pH was also reported.

### 2.5. Equilibrium Study

Sorption experiments and data analysis were carried out on single and binary component systems separately. For the single system, in 25 mL of individual solutions of Hg(II) and Pb(II) of 0.2 mmol·L^−1^ were added 25 mg of ChiFer(III) and agitated for 48 h at 180 rpm in a laboratory orbital shaker. The pH of the solution was adjusted to 4.5, before mixing it with the sorbent material.

The adsorption capacity was calculated by the Equation (1). Furthermore, to investigate the better fitting of the equilibrium parameters, Langmuir [19], Freundlich [20] and Sips [21] models were evaluated according to non-linear Equations (2)–(4) respectively.

Sorption capacity equation:

(1) $$q_{e}=\frac{V(C_{o}-C_{e})}{w}$$

Langmuir equation:

(2) $$q_{e}=\frac{q_{\max}bC_{e}}{1+bC_{e}}$$

Freundlich equation:

(3) $$q_{e}=K_{F}C_{e}^{1/n}$$

Sips equation:

(4) $$q_{e}=\frac{q_{\mathrm{ms}}K_{s}C_{e}^{1/ms}}{1+K_{s}C_{e}^{1/ms}}$$

where *q*_e_ is the amount of metal adsorbed in (mmol·g^−1^), *C*_o_ and *C*_e_ are the initial and equilibrium concentrations respectively in (mmol·L^−1^), *q*_max_ is the Langmuir maximum capacity in monolayer expressed in (mmol·g^−1^), *b* is the Langmuir constant in (L·mmol^−1^), *K*_F_ is the Freundlich constant, *n* is sorption intensity, *q*_ms_ the Sips maximum adsorption capacity (mmol·g^−1^), *K_s_* is the Sips equilibrium constant in (L·mmol^−1^) and *ms* is the Sips model exponent.

For binary component systems, solutions of 25 mL of mixed Hg(II)-Pb(II) at equimolar concentrations of 0.15 mmol·L^−1^ with 25 mg of sorbent material, under the same operation conditions of reaction time and agitation speed (48 h and 180 rpm). In binary component systems, the sorption mechanism can be explained using multi-component models [22], based on Langmuir competitive isotherm (Equation (5)), and the corresponding Equations (6) and (7) were used:

(5) $$q_{e,i}=\frac{K_{i}q_{m}C_{e,i}}{1+\sum_{j=1}^{N}K_{j}C_{e,j}}$$

For Hg(II) equation:

(6) $$q_{e,Hg\left(II\right)}=\frac{K_{Hg\left(II\right)}q_{m}C_{e,Hg\left(II\right)}}{1+K_{Hg\left(II\right)}C_{e,Hg\left(II\right)}+K_{Pb\left(II\right)}C_{e,Pb\left(II\right)}}$$

For Pb(II) equation:

(7) $$q_{e,Pb\left(II\right)}=\frac{K_{Pb\left(II\right)}q_{m}C_{e,Pb\left(II\right)}}{1+K_{Pb\left(II\right)}C_{e,Pb\left(II\right)}+K_{Hg\left(II\right)}C_{e,Hg\left(II\right)}}$$

where, K_1_ and K_2_ are the constants of the model (mmol·g^−1^)

### 2.6. Kinetics

The kinetics parameters were evaluated both in single and mixed solutions; 50 mg of ChiFer(III) were added to each 500 mL in 0.25 mmol·L^−1^ of Hg(II) and Pb(II) solutions (for single experiments), the agitation velocity was kept constant at 180 rpm. Pseudo-first order (PFORE) and pseudo second order (PSORE) models were assessed to fit the experimental data and to obtain the kinetics parameters according to Equations (5) and (6):

Pseudo-first order rate Equation (PFORE):

(5) $$\frac{dq_{t}}{dt}=K_{1}\left(q_{1}-q_{t}\right)$$

Pseudo-second order rate Equation (PSORE):

(6) $$\frac{dq_{t}}{{\left(q_{\mathrm{eq}}-q_{t}\right)}^{2}}=K_{2}dt$$

where *q*_eq_ is the equilibrium sorption capacity (mmol·g^−1^), *q*_t_ is the sorption capacity (mmol·g^−1^) at any time *t* (h) and *K*_2_ is the pseudo-second order rate constant (g·mg^−1^·min^−1^). The parameters *q*_eq_ and *K*_2_ parameters are pseudo-constants.

### 2.7. Salt Effects

The effect on the sorption of Hg(II) and Pb(II) by the presence of sulfate and chloride salts was evaluated. The sulfate concentrations were chosen based on real concentrations of sulfate in wastewater found around gold mining zones (maximum found 223.68 mg·L^−1^ [23]). Twenty milligrams of sorbent material was added to 100 mL of 0.1 mmol·L^−1^ of Hg(II) and Pb(II) binary solutions previously charged with 0.001, 0.05, 0.1 and 0.2 mmol·L^−1^ of sodium sulfate and the initial pH was set at 4.5.

Moreover, a huge range of sodium chloride concentrations were evaluated in the sorption of a binary solution of Hg(II) and Pb(II); these evaluations resembled sodium chloride concentrations normally found in rivers, underground water and seawater. Four different concentrations of NaCl (1.0, 10.0, 100.0 and 500.0 mmol·L^−1^) in 0.1 mmol·L^−1^ of Hg(II) and Pb(II); 20 mg of sorbent material in 100 mL of each solution at pH 4.5 were constantly agitated at 180 rpm for 48 h and the remaining concentrations of Hg(II) and Pb(II) were measured. In order to avoid metal precipitation in the mix solution, a theoretical diagram of chemical species was evaluated; all chemical species diagrams were created using Medusa free software (KTH Royal Institute of Technology, Stockholm, Sweden, version 2013) which are provided in the Supplementary Materials (Figures S1–S3).

### 2.8. Desorption Cycles

To know the possibility of reusing the composite material, sorption–desorption cycles were done in two stages; the first stage, to choose a proper eluent, and the second to assess the reusability across sorption/desorption cycles. In previous sorption/desorption cycles, seven eluents were assessed in one desorption cycle, HNO_3_ (pH = 3.5), HCl (pH = 3.5), NaOH (pH = 11), NaOH (pH = 13), Thiourea 0.1 M (pH = 3.5), Thiourea 0.05 M (pH = 3.5) and EDTA 0.05 M (pH = 10). The binary Hg(II)-Pb(II) solutions were prepared at 0.15 mmol·L^−1^, 25 mg of sorbent material were added to 25 mL of solution at pH = 4.5 for sorption. Then, three sorption–desorption cycles were performed with the selected eluents; the desorption efficiency was calculated according to Equation (7). All tests were made in duplicated.

(7) $$\%\;\mathrm{desorption}=\frac{m_{A}-m_{D}}{m_{A}}\times 100$$

where *m*_A_ and *m*_D_ are the sorbed and eluted mass of the metals (mg) at each sorption/desorption cycle.

## 3. Results and Discussion

### 3.1. Characterization

#### 3.1.1. SEM-EDX Analysis

**Figure 1:**
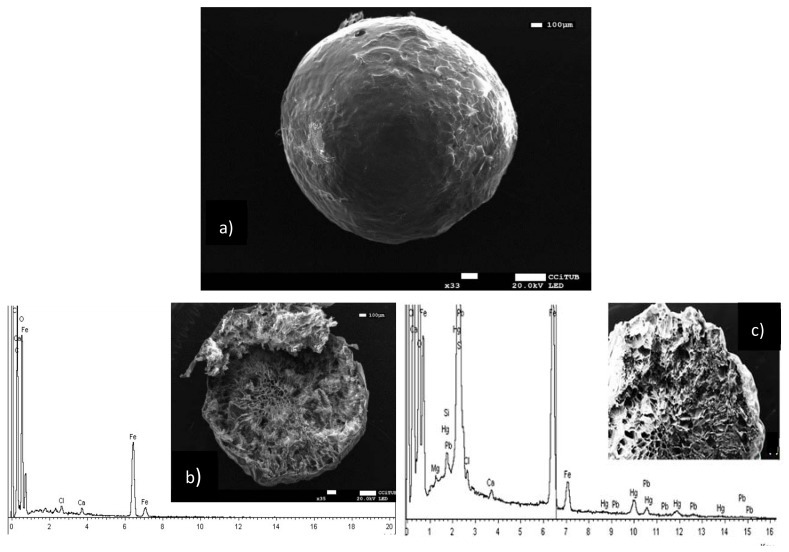
SEM-EDX images. (**a**) ChiFer(III) bead, (**b**) segmented/cross sectional view and EDX of ChiFer(III) bead, (**c**) segmented view and EDX of ChiFer(III) bead after sorption of Hg(II) and Pb(II).

Figure 1 shows the morphology and elemental analysis of the composite material before and after contact with Hg(II) and Pb(II). In Figure 1a the sphericity and roughness of the material is clearly observed.

In Figure 1b a longitudinal slit shows images of the inner porosity, with holes of around 50–100 µm, which can be compared with glutaraldehyde-cross-linked-chitosan-spheres reported by [24] in terms of porosity and size. In addition, in the same Figure 1b EDX analysis shows the presence of iron(III) in its structure. Furthermore, the addition of iron(III) does not affect the inner morphology of the beads. The beads are observed after sorption of Hg(II) and Pb(II) in Figure 1c. The porosity inside the composite did not change. The EDX analysis confirmed the presence of Hg(II) and Pb(II) as a result of the sorption process.

#### 3.1.2. FTIR and TGA Analysis

**Figure 2:**
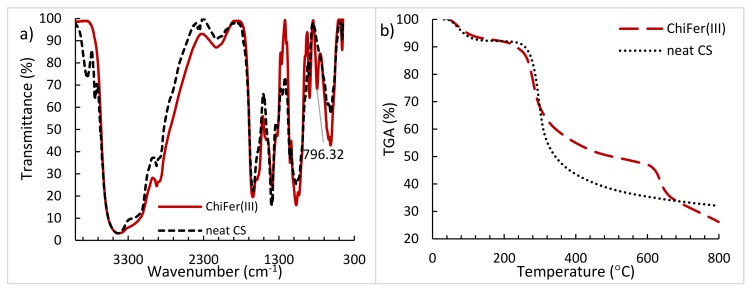
(**a**) FTIR and (**b**) TGA curves of neat CS and ChiFer(III).

Figure 2 shows the FTIR spectra (Figure 2a) and TGA analysis (Figure 2b) of neat chitosan (neat CS) and chitosan-iron(III) composite ChiFer(III). In FTIR both samples show a broad band at 3400–3600 cm^−1^ (hydroxyl groups) for neat CS and stretching vibrations and Fe–OH for ChiFer(III), C–H stretching vibration at 2925 cm^−1^ and O–H group of polysaccharides in 1639 cm^−1^ in neat CS [25,26], and slight change at 1644 cm^−1^ for ChiFer(III). As Sipos et al. [27] reports, well know bands of FeOOH are 1620 cm^−1^, 1500 cm^−1^ and 1340 cm^−1^, the first two peaks are overlapped in the ChiFer(III) specter, while the latter peak at 1384 cm^−1^ is well noted. The more notable difference in both spectra is the peak at 796 cm^−1^ in ChiFer(III), which is attributed to iron species reported by Ruan et al. [28]. The major iron species are overlapped in the main characteristic bands of neat chitosan, which could indicate the good cohesion of the composite. Also, FTIR analysis were performed before and after sorption (provided in Supplementary Materials Figure S4). In ChiFer(III) material the mean peak of amino and hydroxyl groups (3400 cm^−1^) are overlapped by the presence of iron. Besides, after Hg(II) and Pb(II) sorption, the FTIR specter present the same behavior, it is to say that the metals were mainly bound by remaining amine groups, particularly at stretching vibrations of 3400 cm^−1^, 1631 cm^−1^ (C = N bond), 1376 cm^−1^ and 1073 cm^−1^ peaks correspond to stretching vibration of C–OH. Moreover, in this study was not evidenced the typical peaks of 580 cm^−1^ and 759 cm^−1^ of Fe–O complex (indicative of metal-metal complexes).

Additionally, TGA analysis of neat CS and ChiFer(III) were conducted. The results of both samples are showed in Figure 2b; a first weight loss at 90 °C is produced due to the volatilization of low-molecular weight compounds, such as water [29]. The drop of the curves between 250–300 °C is related to the oxidation and degradation of chitosan, this is in concordance with Yu et al. [30]. Above 300 °C is observed a significant difference of loss weight for CS and ChiFer(III), it can be attributed to the thermal degradation of the polysaccharides chains of chitosan. According to Hong et al. [29], the kinetic decomposition follows the Ozawa–Flynn–Wall method [31].

It is noteworthy that the amount of iron into the composite corresponds to 33% (dried weight, d.w.), so the 67% is neat chitosan; evidently, for 6 mg samples ChiFer(III) material has less chitosan content than pure CS samples. Between 300–600 °C the weight loss of chitosan is higher than ChiFer(III), as expected (i.e., the weight loss for chitosan is 64% and for ChiFer(III) is 52%); it means that the incorporation of iron into the chitosan matrix provides high thermal stability to the resulting material (at temperatures between 300–600 °C). However, at 600 °C occurs a steep decomposition of ChiFer(III) which is attributed by Ziegler et al. [32] to the conversion of the inorganic core of iron(III). The iron species can be converted to magnetite, maghemite or wüstite Fe1-xO, consequently a weight loss is produced, which is accompanied with the evaporation of volatiles sub-products. This complex phenomenon depends on the structure and binding species in the composite. However, in sorption applications, such temperatures are not reached, moreover, TGA analysis in this study enhances the knowledge of the thermal stability of the materials.

### 3.2. pH Study

The pH plays a transcendental role when it is used in chitosan-based composites in metal sorption procedures [33]. Sorption experiments at different pHs were evaluated from pH = 2.0 to pH = 7.0 in single and binary solutions. It is noteworthy that at pH < 3.0 the ChiFer(III) composite is less stable (due to the chitosan hydrolysis and the consequent dissolution of the organic and inorganic content in the beads), while pH > 6 insoluble hydroxide precipitates would be formed (the images of the material stability at different pHs are provided in Supplementary Materials (Figure S5)). All these considerations were taken into account when performing the experiments and to avoid the precipitation phenomenon.

**Figure 3:**
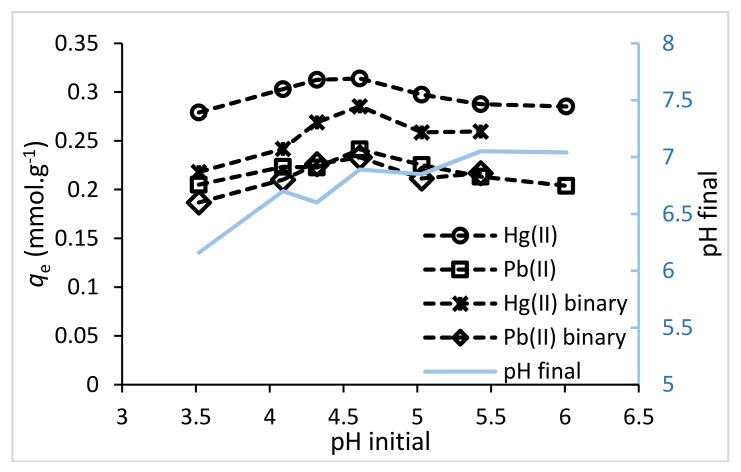
Adsorption capacity at different pHs. (*T*: 20 °C; sorbent dosage: 1 g·L^−1^; agitation speed: 180 rpm; contact time: 48 h; *C*_0(single)_: 0.2 mmol·L^−1^; *C*_0(binary)_: 0.1 mmol·L^−1^).

Figure 3 shows the adsorption capacity at various pHs. The best pH observed was between 4.5 and 5.0. This accords with other researchers [34,35,36] who tested chitosan-based composites. It is remarkable that Hg(II) sorption is greater than Pb(II) sorption in both single and binary component systems. In binary system, it is clearly shown the variations in pH, which do not surpass pH 7 (due to the buffer effect of chitosan).

**Figure 4:**
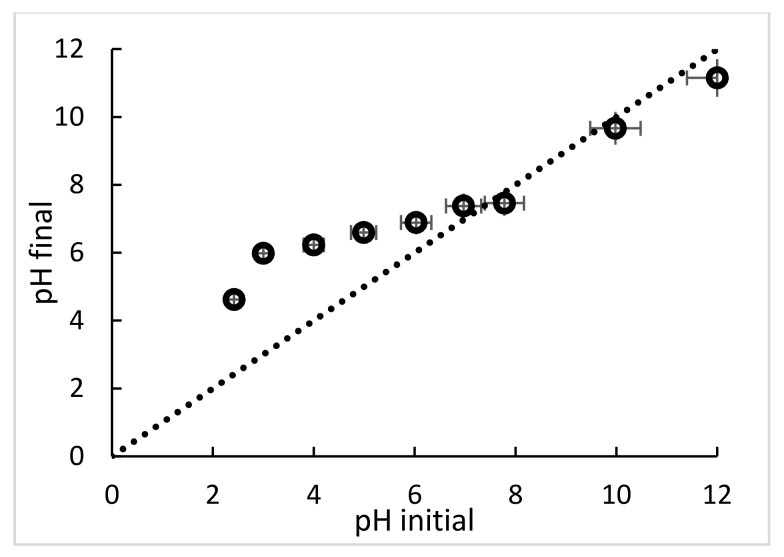
pH_pzc_ of ChiFer(III). (*T*: 20 °C; sorbent dosage: 1 g·L^−1^; agitation speed: 180 rpm; contact time: 24 h; 0.01 M NaCl solution).

However, the pH where the majority of surface sites are neutral, and the net charge on the surface is zero, is known as the point of zero charge (pH_pzc_) [37], this value was evaluated and is presented in Figure 4. Zero net surface charge density does not imply the absence of any charges, but rather the presence of equal amounts of positive and negative charge [38]. In general, ligand exchange is favored at pH levels less than the pH_pzc_ [39], as below pH_pzc_ more sites are able to be protonated or, failing that, be able to be occupied by cations, depending on the predomination of electrostatic forces or chelating bonding [35]. The pH_pzc_ of ChiFer(III) was recorded at 7.40 which is depicted in Figure 4, this is in agreement with findings in the literature [40]. The pH_pzc_ of neat chitosan was reported as 7.1 and pH_pzc_ of Fe(OH)_3_ was 6.9 [38]. Consequently, the pH_pzc_ value of ChiFer(III) corresponds somewhere between neat chitosan and its ferric form.

The optimal pH for Hg(II) and Pb(II) sorption, match with the pH values under pH_pzc_. These accords with several authors who reported behaviour below pH_pzc_ for Hg(II) [41] and Pb(II). Moreover, the main variations in pH were observed between pH > 3.0 < pH_pzc_, due to the protonation of the amino groups into the ChiFer(III) material. This means that in this pH range a competition between metals and protons for the active sites in the sorbent could be produced; which is confirmed by the “buffering effect” at pH 4–6 (Figure 4). Consequently, the sorbate/sorbent interactions to these systems are mainly based on chelation bonding of metals with nitrogen atoms and hydroxides iron species, and to a lesser extent on electrostatic interactions. The optimum operational pH was found as pH 4.5. Henceforth, the experiments were performed at this initial pH.

### 3.3. Equilibrium

**Figure 5:**
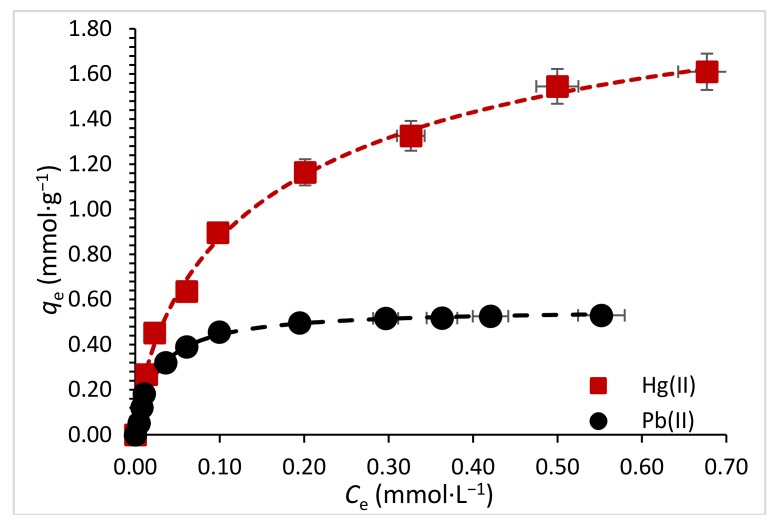
Single component sorption isotherms. (*T*: 20 °C; sorbent dosage: 1 g·L^−1^; agitation speed: 180 rpm; contact time: 48 h).

**Figure 6:**
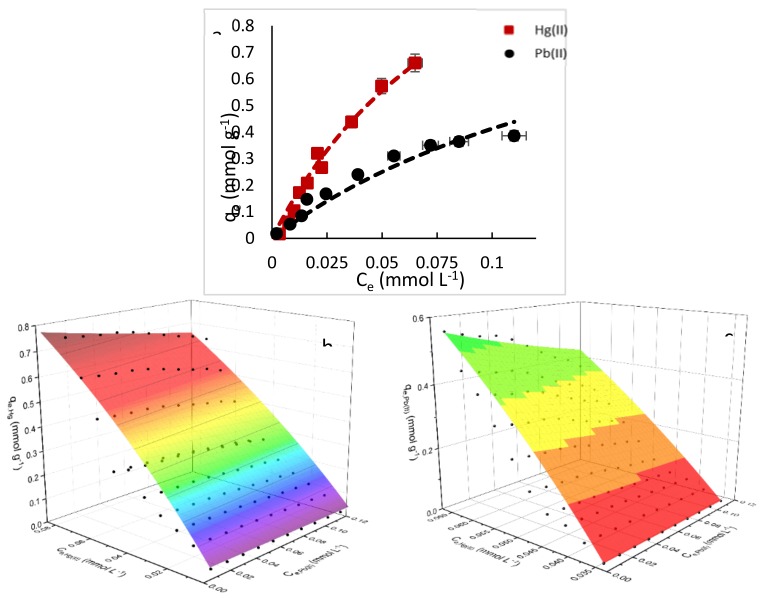
(**a**) Binary component sorption isotherms, (**b**) 3D surface for Hg(II) sorption capacity response vs. Hg(II) and Pb(II) interactions, and (**c**) 3D surface for Pb(II) sorption capacity response vs. Hg(II) and Pb(II) interactions. (*T*: 20 °C; sorbent dosage: 1 g·L^−1^; agitation speed: 180 rpm; contact time: 48 h, pH_o_ = 4.5, *C*_o_ = 0.15 mmol·L^−^^1^).

The correlation of data by theoretical Equations is fundamental for the engineering design and scaling-up of sorption systems. Several models are proposed in the literature for fitting the experimental data, it includes Langmuir, Freundlich and Sips Equations. This fitting does not mean that the principles of the models are verified, but it could improve the interpretation of the sorption mechanisms [42]. The impact of metal concentration on sorption uptake is demonstrated by a progressive increase until a saturation plateau is reached. Figure 5 and Figure 6 show the equilibrium data for Hg(II) and Pb(II) for single and binary component systems respectively, from aqueous solutions at initial pH of 4.5 and 20 °C. The obtained parameters of the models for single component are summarized in Table 1.

In single component, Sips model shows the best fitting in terms of *r*^2^, however it presents a large standard error in the *K*_s_ parameter. Likewise, the Langmuir model gets good *r*^2^ > 0.98, but much less standard (Std.) error values than the Sips model. Therefore, Langmuir model is taken as reference for the interface analysis. Nonetheless, the characteristic asymptotic shape of the isotherm is consistent with the Langmuir Equation. The maximum sorption capacity (*q*_max_) of Hg(II) and Pb(II) were 1.80 and 0.56 mmol·g^−1^, respectively. Thus, the sorption capacity for mercury ions is three times higher than that for lead ions.

In terms of sorption capacity performance, ChiFer(III) is competitive compared with other chitosan composites; e.g., Dhanapal et al. [43] tested acryloylated chitosan, 2-acrylamido-2-methyl-1-propansulfonic acid, 2-(diethylamino) ethylmethacrylate and *N*,*N*′-methylene bisacrylamide as a crosslinker (ACAD), obtaining a sorption removal of 2.26 mmolHg(II)·g^−1^. Similarly, Zhang et al. [44] manufactured the cobalt ferrite/chitosan grafted with graphene composite (MCGS) material and the sorption capacity obtained was 0.67 mmolHg(II)·g^−1^.

In the case of Pb(II), chitosan/magnetite [45], and thiolated chitosan [12] materials were tested, and the sorption uptake was in the order of 0.30–0.53 mmolPb(II)·g^−1^. Table 2 shows additional studies regarding to chitosan-based composites for single sorption of Hg(II) and Pb(II). It is noteworthy that the ChiFer(III) material is configured in the form of beads, and this could contribute to the scale-up for future industrial manufacturing.

Regarding to the sorption affinity, it is clearly seen that Hg(II) ions are more sorbed onto ChiFer(III) material than Pb(II) ions: Hg(II) > Pb(II). This could be explained by the ionic radii differences between Hg(II) and Pb(II), which is 1.02 and 1.19 respectively. Therefore, Hg(II) ions can enter into the material pores easier than Pb(II) ions. However, more than one mechanism is presented to explicate in deep how the metals are bonded onto the material. This affinity accords with that reported by Zhu et al. [46], who carried out sorption experiments with Hg(II) and Pb(II) onto chitosan with thiourea groups where the sorbent had more affinity towards Hg(II) than to Pb(II) ions. Several experiments with the raw chitosan particles were carried out in single system (with 0.2 mmol·L^−^^1^ solutions of Hg(II) and Pb(II)) for comparing the sorption efficiency of ChiFer(III) beads. Figure S6 shows that the sorption capacity for Hg(II) is almost similar for both materials (i.e., 1.02 mmol·g^−1^ for chitosan and 0.90 mmol·g^−1^ for ChiFer(III)); the performance of chitosan particles is slightly higher than ChiFer(III); this can be attributed to the small particle size of chitosan (<0.5 mm), which in comparison with ChiFer(III) beads (2 mm), has a greater impact on the resistance to the film diffusion; making the active sites of chitosan easier accessible for mercury ions. In addition, ChiFer(III) material is more efficient for lead removal than chitosan at the same operation conditions (i.e., 0.2 mmol·g^−^^1^ for chitosan and 0.45 mmol·g^−^^1^ for ChiFer(III)); it means that the introduction of hydroxyl groups of iron(III) hydroxide improves the sorption uptake of metal ions and could improve the stability of the resulting beads [17].

Many published results in the literature are based in single systems, however the evaluations of the binary components had more interest for future applications in real effluents [49]. In this present study, the equilibrium analysis was done under the concept that one binding site was only available for one sorbate, supported by the competitive Langmuir isotherm model, according to Equation (5) and combination of Equations (6) and (7), which were simultaneously solved using Origin 9.0 software (OriginLab Inc., Northampton, MA, USA, 2012). Many authors have taken into account the simple models and have omitted the competitive effect on secondary species. Thus, recent publications reported the use of the competitive equilibrium Equations [50,51,52].

The constant values of the bi-component model were *K*_Hg(II)_ = 5.68, *K*_Pb(II)_ = 2.24 and *q*_m_ = 2.87 as shown in Table 1. Furthermore, the determination coefficient was acceptably fitted at *r*^2^=0.95. Figure 6 illustrates the isotherms related to the competition of Hg(II) and Pb(II) at pH_o_ = 4.5 and the same initial concentrations of 0.15 mmol·L^−1^. It is noted that the material has the same trend as in single systems. In other words, the affinity stays stronger in Hg(II) ions rather than in Pb(II) ions. To better illustrate of the two metal ions on the sorption capacity of each metal ion, 3D surfaces (as seen in Figure 6b), which shows a more marked decrease as the concentration of Pb(II) ions increase.

**Figure 7:**
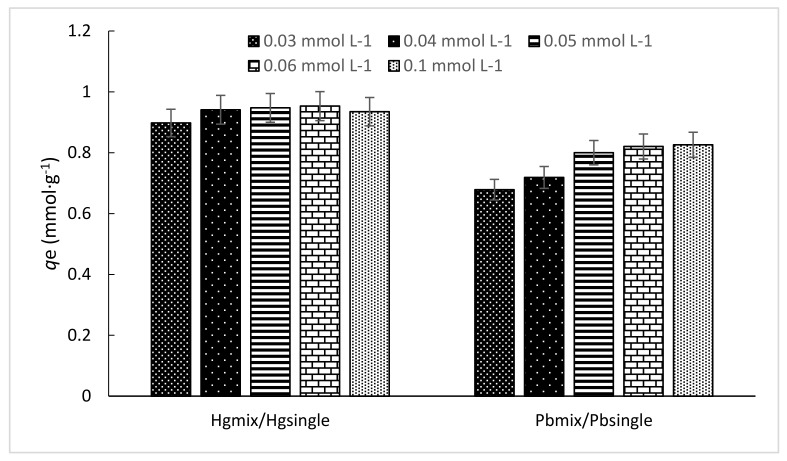
Sorption capacity ratio (scr) changes. (*T*: 20 °C; sorbent dosage: 1 g·L^−1^; agitation speed: 180 rpm; contact time: 48 h, pH_o_ = 4.5, *C*_o_ = 0.03–0.1 mmol·L^−1^).

According to Mohan et al. [49], the effect of ionic interactions on sorption may be represented by the ratio of the sorption capacity for one metal ion in the presence of another metal ion (*Q*^mix^), to the sorption capacity for the same metal when it is present (*Q*^0^). Although this relation was used with *Q*^0^ = *q*_max_ (for this nomenclature) and calculated by models. In this work we used the same relation for an equilibrium concentration at a single point in the isotherm *q*_e_ instead of *q*_max_, as it is not possible to reach the isotherm plateau due to the imminent precipitation at concentrations over 0.15 mmol·L^−1^ at pH = 5.5. It is also possible to note the ratio differences at different equilibrium concentrations. Thus, the effect of ionic interactions by each metal is calculated by the sorption capacity ratio (scr), scr = *q*_emix,i_/*q*_e,i_; so, for Hg(II) the scr_Hg(II)_ = *q*_emix,Hg_/*q*_e,Hg_ and scr_Pb(II)0_ = *q*_emix,Pb_/*q*_e,Pb_. Figure 7 show the scr behaviour in both metals at different *C*_e_ concentrations. It is noted that difference between the ratio is not marked in Hg(II) ions as scr changes, which means that although Pb(II) ions are in the solution, these ions do not suppress the sorption of Hg(II) ions. On the other hand, Pb(II) ions capacity decreases as long as the *C*_e_ increases, which means that there is a suppression of Pb(II) ions is achieved towards the isotherm plateau is achieved. It also indicates preference for Hg(II) ions in this Hg(II)-Pb(II) system.

### 3.4. Kinetics Studies

**Figure 8:**
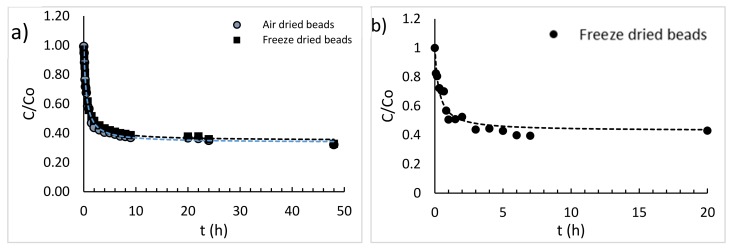
Kinetics profiles: (**a**) Hg(II), (**b**) Pb(II). (*T*: 20 °C; sorbent dosage: 1 g·L^−1^; agitation speed: 180 rpm; contact time: 48 h; *C*_0_: 0.2 mmol·L^−1^; dashed line: fitting with pseudo-second order rate equation).

The sorption kinetics studies are important for determining the required contact time of the sorbate/sorbent systems. In industrial applications the time for achieving the maximum saturation plateau is a very relevant parameter for design of reactors [53]. Figure 8a,b show the kinetic profiles for lead and mercury from aqueous solutions. It is noteworthy that the kinetic uptakes are controlled by different mechanisms including: (i) bulk diffusion; (ii) external diffusion (so-called film diffusion); (iii) intraparticle diffusion; and (iv) reaction rate. Demey et al. [17] reported that maintaining a continuous agitation speed of 150–200 rpm (and a sorbent dosage of 1 g·L^−1^) is enough to avoid the settling of the sorbent and neglecting the contribution to the bulk diffusion.

The kinetics studies of mercury (Figure 8) were performed with two types of drying beads configurations (i.e., freeze-dried beads, which is the standard material synthesized in this work, and the air dried beads). A comparison of the drying method was performed to evaluate the accessibility of the metal species into the porous network of the sorbent. Both curves shown in Figure 8 are overlapped, confirming not significant influence of the drying techniques on the metal uptake. These surprising results are in contradiction with those obtained recently by Demey et al. [17], in which ChiFer(III) material for neodymium recovery from aqueous solutions, and the accessibility of the metal into the active sites was affected according to the drying method and, as a consequence, the equilibrium time was impacted.

In this work, important differences between the drying techniques were not found, probably due to several causes: (i) the structure of the polymeric matrix remained relatively open after air drying and it did not affect the accessibility into the pores (this was not completely evidenced in the SEM images of the composite); (ii) the covalent radius of neodymium is bigger than that of the mercury ions, and these latter ions had not difficulties in transporting into the entrance of the pores (i.e., the covalent radius of mercury is 132 pm and the covalent radius of neodymium is 201 pm). Rorrer et al. [54] concluded that a pore blockage may occur at low metal concentrations. The migration of sorbed species is low, which in conjunction with the larger size of the neodymium ions, results in an accumulation at the entrance of the pore. Therefore, the low-cost air drying technique does not have strong influence in the sorption of mercury. This opens the door to manufacturing the materials on a higher scale so as to conduct future evaluations with real industrial effluents.

Regardless of the metal, (mercury or lead), the curves in Figure 8a,b are characterized by three progressive pseudo-steps: (i) an initial step that takes around 50–60 min; (ii) a second step that takes around 4–5 h, and (iii) a third step that takes around 60 min. The differences of each step are related to the gradient mass that is progressively reduced as a function of the contact time. Table 3 reports the comparison of the experimental sorption capacities at equilibrium with calculated values for both pseudo-first order (PFORE) and the pseudo-second order rate equations (PSORE). The correlations confirm much better fitting with PSORE.

### 3.5. Salt Effects

**Figure 9:**
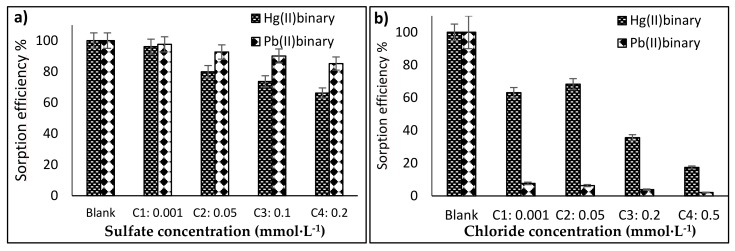
Salt effects: (**a**) Sulfate effect, (**b**) Chloride effect. (*T*: 20 °C; sorbent dosage: 0.2 g·L^−1^; agitation speed: 180 rpm; contact time: 48 h; *C*_0_: 0.1 mmol·L^−1^).

Figure 9a,b show the sorption behaviour when sodium sulfate and sodium chloride are added in binary Hg(II)-Pb(II) systems. In the case of sulfate ions, the material sorption capacity slightly decreases as the sulfate concentration increases. This effect is more pronounced in Hg(II) than in Pb(II) ions. However, it is more noticeable that even at high sulfate concentration (0.2 mmol·L^−1^), the sorption capacity decreases at around 35% and 15% for Hg(II) and Pb(II) respectively. This means that the material sorption capacity was lightly affected by the sulfate ions, which in turn is beneficial for the treatment of wastewater.

The chloride effect in sorption efficiency of the material was then assessed, covering weak and strong chloride concentrations (0.001 M similar to (s.t.) potable water, 0.05 M s.t. river water, 0.2 M s.t. underground water and 0.5 M s.t. seawater). In all cases, sodium chloride suppresses the sorption capacity, even at low chloride concentrations. The sorption of Pb(II) was affected more strongly than the Hg(II) ones, around 97–98% and 40–83% for Pb(II) and Hg(II) respectively. This can be explained as follows: the formation of complexes with chloride ions is easier than sulfate ions [55]; i.e., chloride may form complexes with Pb(II) and Hg(II). According to Kinniburgh et al. [56] who studied the adsorption of Hg(II) on Fe gel, found that in the presence of chloride the adsorption of Hg(II) is considerably reduced. However, the biggest suppressing affect was on the Pb(II) sorption, which could indicate that the chloride-complex is more easily formed with Pb(II) rather than Hg(II). On the other hand, this factor could impact on the selectivity of the material, which in the presence of chloride, the Hg(II) is successfully adsorbed but the Pb(II) sorption has been substantially reduced (Figure 9b).

Comparing the effects of sulfates and chlorides on the sorption of Hg(II) and Pb(II), it is noted that chlorides suppress the adsorption much more than sulfates. This is in agreement with Mitani et al. [57] who found that sulfates were adsorbed in greater quantities than chlorides when this act as counter ions with metals over chitosan-based gel. That is to say that sulfates have less effect on sorption than chlorides with chitosan. In this work we found the affinity over our iron/chitosan composite as: Hg(II) > Pb(II).The possibility of regeneration, as well as the recover.

### 3.6. Desorption

**Figure 10:**
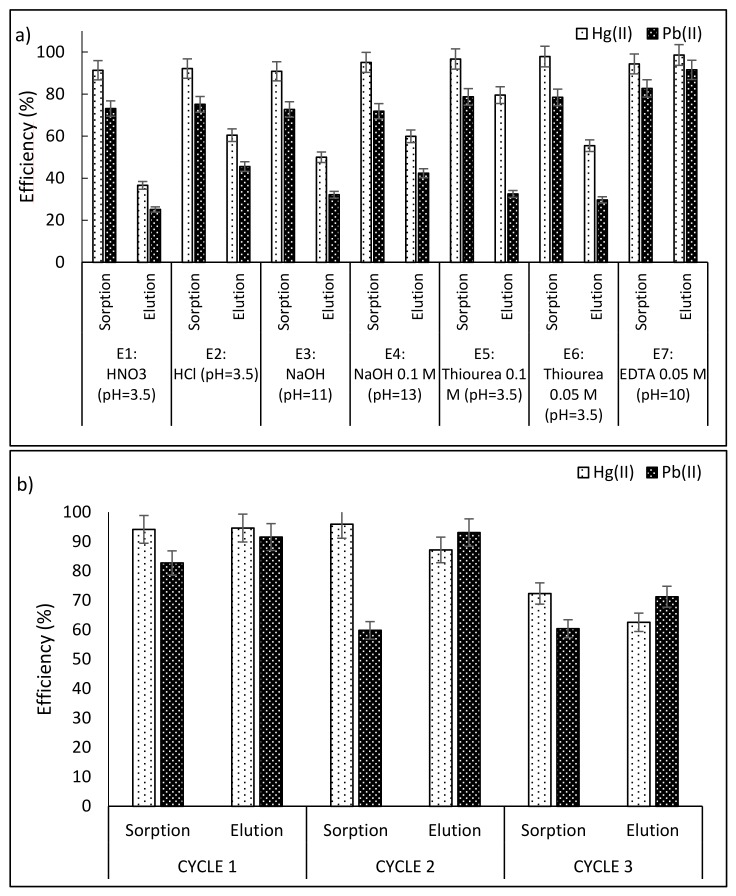
Desorption: (**a**) Eluent selection, (**b**) Cycles with the selected eluent: EDTA solution pH 10. (*T*: 20 °C; sorbent dosage: 1 g·L^−1^; agitation speed: 180 rpm; contact time: 24 h; *C*_0_: 0.15 mmol·L^−1^).

The possibility of regeneration, as well as the recovery of metals from the loaded sorbent, is an important parameter for evaluating the feasibility of the sorption processes. In the first stage, seven eluents in one sorption/desorption cycle were tested, the results of which are illustrated in Figure 10a. The eluents were: HNO_3_ (pH = 3.5), HCl (pH = 3.5), NaOH (pH = 11), NaOH (pH = 13), Thiourea 0.1 M (pH = 3.5), Thiourea 0.05 M (pH = 3.5) and EDTA 0.05 M (pH = 10). Eluents HNO_3_, HCl, NaOH and thiourea 0.05 M shows recoveries below 60% and 50% for Hg(II) ions and Pb(II) ions respectively. HCl at pH = 3.5 represent a good eluent, this is in concordance with the Section 3.5 of this article, which describes the salt effects, the impact of protons was verified, as a result of a higher concentration of H^+^, a strong competition for the active sites is produced, and this effect is taken in advantage for desorption procedures. Moreover, thiourea 0.1 M present 80% of recovery for Hg(II), but 32% of elution recovery for Pb(II) ions in the first sorption/desorption cycle, but after this, thiourea directly impacts on the stability of the sorbent and the beads become mechanically fragile; thus, their original brown color turns to green. It means that thiourea acts as a reducing agent and consequently iron(III) is reduced to iron(II); it is in agreement with the findings of Zhu, 1992 [58] who reported that the redox interactions of ferric ions and thiourea follows a first order reaction.

Nevertheless, EDTA 0.05 M set at pH 10 achieved recoveries of 98% for Hg(II) and 91% for Pb(II), so, in order to assess the performance of reusability three sorption/desorption cycles were carried out with EDTA 0.05 M, which is illustrated in Figure 10b.

Along three sorption/desorption cycles, alkaline EDTA shows excellent recovery for both metals, with average elution efficiencies of 81.43% for (Hg(II)) and 81.43% for (Pb(II)), it was up to 87% for the first two cycles, followed a lightly drop in the third cycle with recoveries of 62.54% and 71.23% for Hg(II) and Pb(II) respectively. Mass of the sorbed and eluted metals of the three sorption/desorption cycles are provided in Figure S7 of Supplementary Materials section. The stability of the beads after the third cycle was good, mainly due to the alkaline media, in which the hydrogel is more stable. Desorption studies reported by [59,60,61], who used EDTA for desorption of heavy metals showed comparable results with the obtained in this study, EDTA at alkaline medium is deprotonated, which in conjunction with its chelating properties, represents an excellent desorbent for heavy metals strongly bonded onto the sorbent matrix.

## 4. Conclusions

ChiFer(III) bio-based sorbent showed good sorption capacity towards the removal of Hg(II) and Pb(II) ions in single and binary systems. There was remarkable affinity for Hg(II) even in strong chloride conditions. Besides, the stability and the performance of the material was maintained during all three sorption–desorption cycles. Langmuir and competitive Langmuir models fitted the equilibrium in single and binary component systems, respectively. The maximum sorption capacities were 1.8 mmol·g^−1^ and 0.56 mmol·g^−1^ for Hg(II) and Pb(II), respectively. Pseudo second order rate equation adjusted accurately to kinetics data. The main advantage of using this material is the very simple manufacturing procedure and cheap cost, added to the high sorption capacity compared with existing similar bio-materials.

## Figures and Tables

**Table 1:** Single and binary component isotherm constants of ChiFer(III) material.

**Single Component System**						
			**Hg(II)**		**Pb(II)**	
	**Parameter**	**Unit**	**Value**	**Error**	**Value**	**Error**
**Langmuir**	***q*_exp_**	(mmol·g^−1^)	1.61		0.52	
	***q*_max_**	(mmol·g^−1^)	1.80	0.06	0.56	0.007
	***b***	(L·mmol^−1^)	10.17	1.31	38.8	2.49
	***r*^2^**		0.988		0.996	
	***q*_max_ × *b***	(L·g^−1^)	18.3		21.44	
**Freundlich**	***K*_F_**	(mmol^1−1/n^·g^−1^·L^1/n^)	1.97	0.07	0.69	0.05
	***n***		2.63	0.19	3.66	0.54
	***r*^2^**		0.982		0.91	
**Sips**	***q*_max_**	(mmol·g^−1^)	2.3	0.26	0.55	0.01
	***K*_s_**	(L·mmol^−1^)	3.14	1.18	52.41	15.04
	***n*_s_**		1.4	0.15	0.93	0.05
	***r*^2^**		0.995		0.998	
**Binary Component System**						
			**Value**	**Error**		
**Langmuir competitive model**	*K*_m_	(mmol·g^−1^)	2.87	0.57		
	*K*_1_	(mmol·g^−1^)	5.68	1.60		
	*K*_2_	(mmol·g^−1^)	2.24	0.63		
	*r*^2^		0.96			

**Table 2:** Adsorption capacity of Hg(II) and Pb(II) with different chitosan-based materials.

Modification	Metal	pH	*T* (°C)	*q*_max_ (mmol·M^+^·g^−^^1^)	Isotherm fitting	Ref.
Microspheres chitosan grafted with chlorosulfonic acid (CSSULF) or ethylenimine (CSPEI)	Hg(II)	6	-	0.32	Langmuir/Freundlich	[35]
Cross-linked aminated chitosan beads	Hg(II)	7		2.23	Langmuir	[33]
Chitosan/Graphene oxide imprinted Pb^2+^	Pb(II)	5	30	0.38	Langmuir	[47]
Polyaniline grafted cross-linked chitosan beads	Pb(II)		45	0.55	Langmuir	[48]
Present study: ChiFer(III)	Hg(II) Pb(II)	4.5	Room	1.80 0.56	Langmuir	

**Table 3:** Kinetic parameters of sorbent materials.

Experimental			PFORE			PSORE		
Metal	Sorbent	*q*_exp_ (mmol·g^−1^)	*K*_1_ (h^−1^)	*q*_1_ (mmol·g^−1^)	*r*^2^	*K*_2_ (g∙mmol^−^^1^∙h^−^^1^)	*q*_2_ (mmol·g^−1^)	*r*^2^
**Hg(II)**	Air dried beads (AD)	1.71	1.44	1.57	0.983	1.18	1.69	0.991
	Freeze dried beads (FD)	1.70	1.36	1.53	0.977	1.12	1.65	0.995
**Pb(II)**	Freeze dried beads (FD)	0.64	2.09	0.63	0.930	4.94	0.679	0.945

## Supplementary Materials

The following are available online at http://www.mdpi.com/2073-4360/10/4/367/s1, Figure S1: Species diagram system of Hg(II), Figure S2: Species diagram system SO_4_^2−^, Hg(II), Pb(II), Figure S3: Species diagram system Cl^−^, Hg(II), Pb(II), Figure S4. FTIR spectres before and after metals sorption, Figure S5: Stability of sorbent material at different pHs, Figure S6: Metal species removal by neat chitosan and ChiFer(III) as sorbents and Figure S7: Sorbent and eluted mass in sorption/desorption cycles.
