# Supplementary material for: Sorption of Hg(II) and Pb(II) Ions on Chitosan-Iron(III) from Aqueous Solutions: Single and Binary Systems
Source: Polymers (Basel). 2018 Mar 24;10(4):367. doi: 10.3390/polym10040367 (PMC6414923; doi:10.3390/polym10040367)
Supplement: Supplementary file 1 [file polymers-10-00367-s001.docx]

Supplementary Materials

Figure S1. Species diagram system of Hg(II)

Figure S2. Species diagram system SO4^2-^, Hg(II), Pb(II)


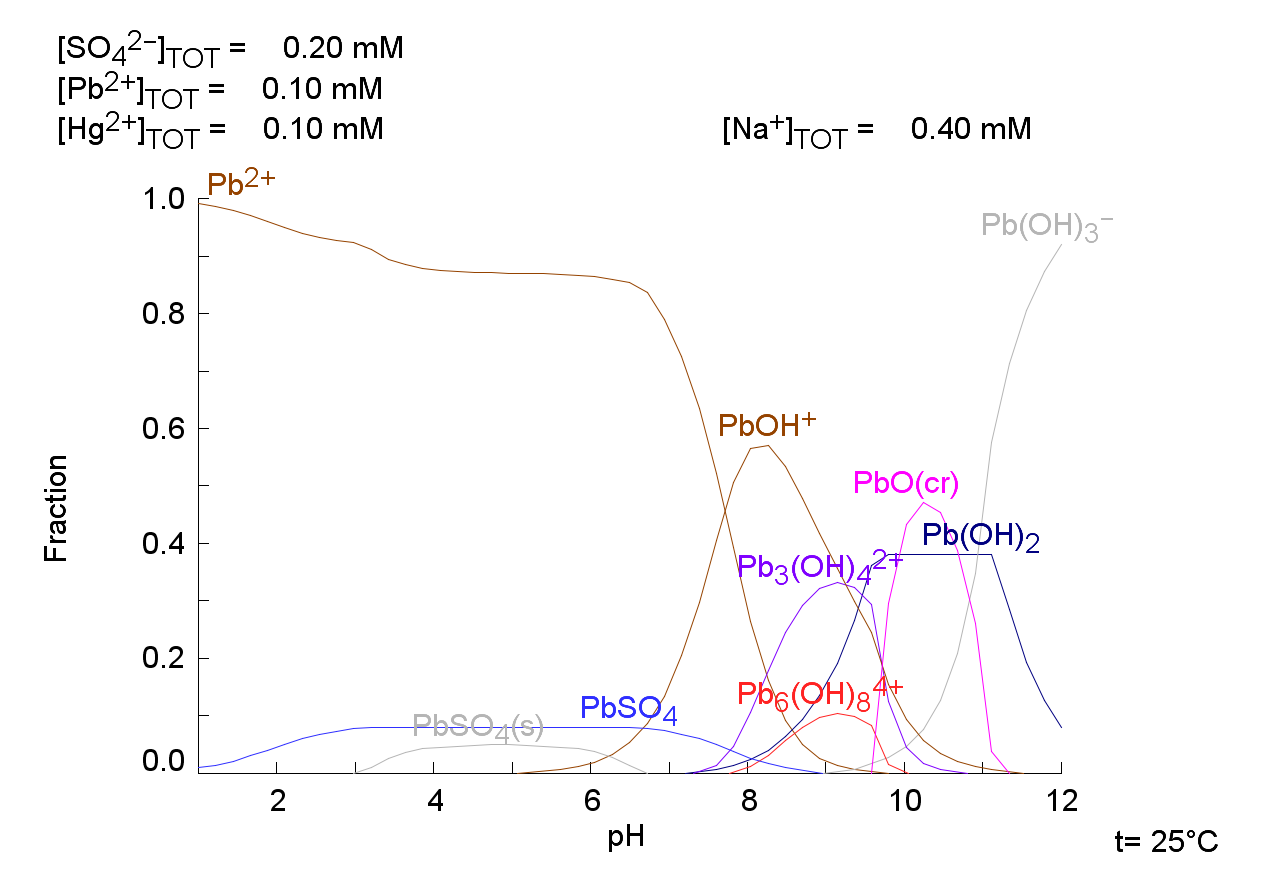


Figure S3. Species diagram system Cl^-^, Hg(II), Pb(II)


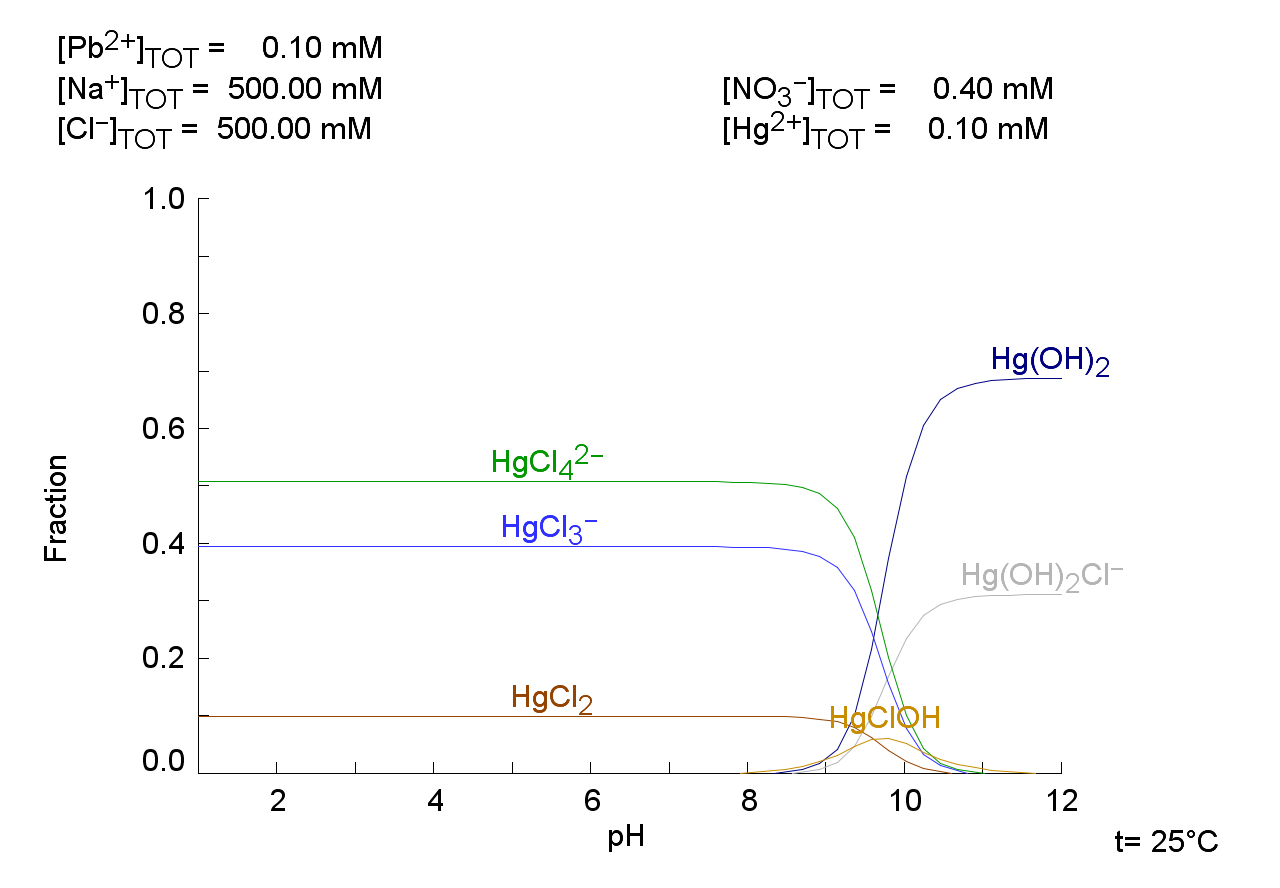


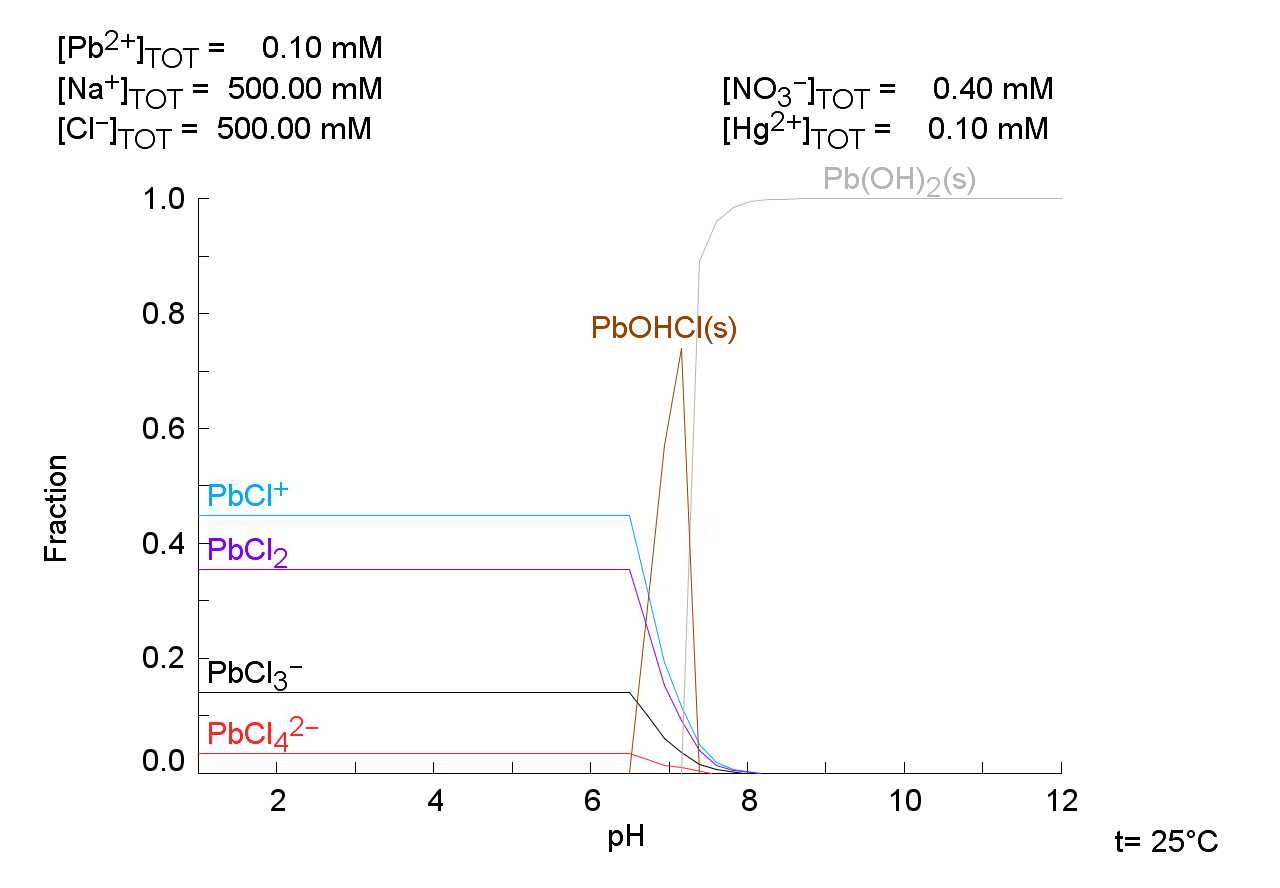


Figure S4. FTIR spectres before and after metals sorption.

Figure S5. Stability of sorbent material at different pHs


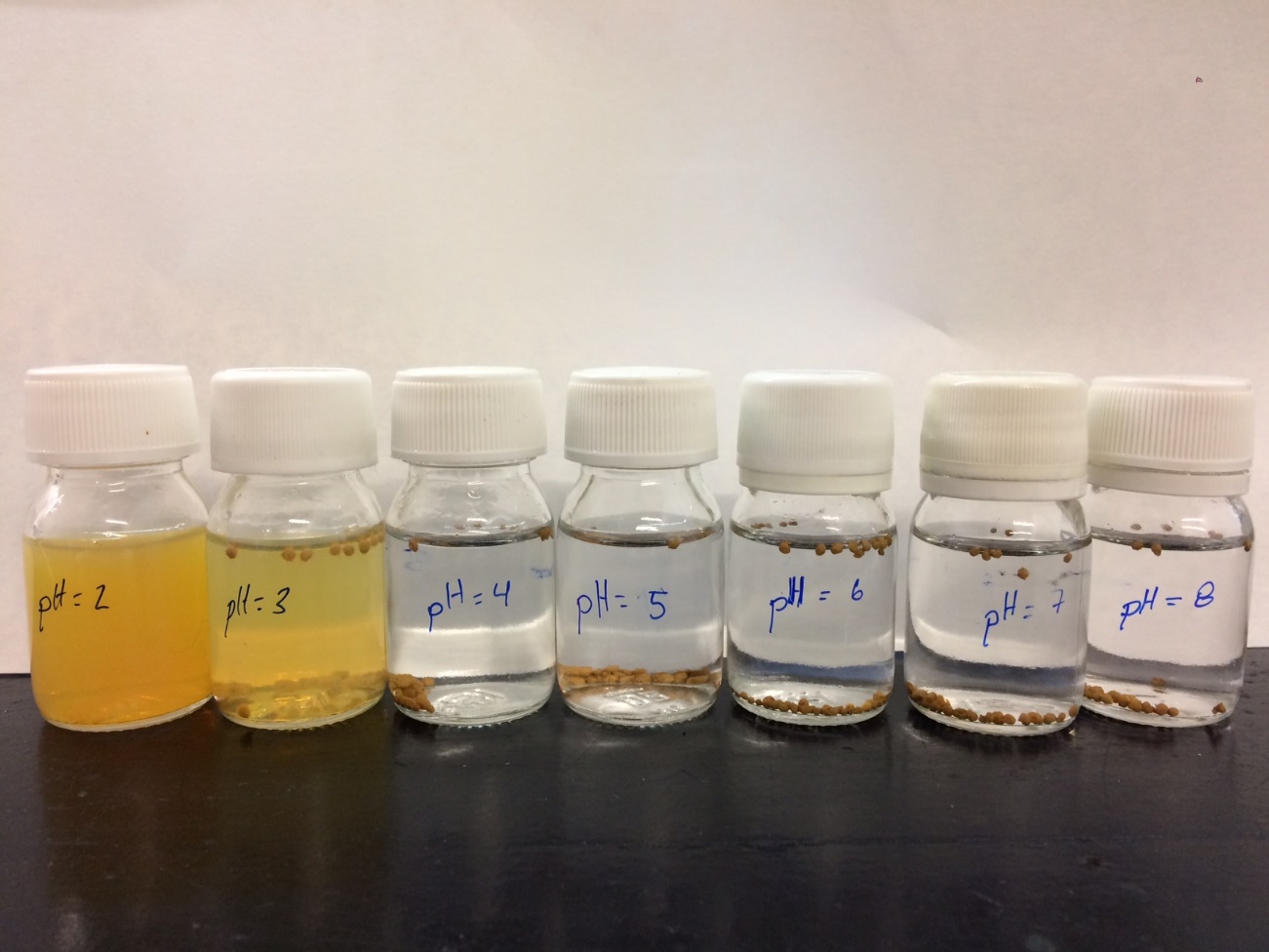


Figure S6. Metal species removal by neat chitosan and ChiFer(III) as sorbents.

(T:20 °C; sorbent dosage: 1 g L^-1^; agitation speed: 180 rpm; contact time: 48 h, Co: 0.1 mmol L^-1^).

Figure S7. Sorbent and elution mass in sorption/elution

(T:20 °C; sorbent dosage: 1 g L^-1^; agitation speed: 180 rpm; contact time: 48 h, Co: 0.15 mmol L^-1^).
